# Autologous Bone-Marrow Mesenchymal Stem Cell Implantation and Endothelial Function in a Rabbit Ischemic Limb Model

**DOI:** 10.1371/journal.pone.0067739

**Published:** 2013-07-04

**Authors:** Shinsuke Mikami, Ayumu Nakashima, Keigo Nakagawa, Tatsuya Maruhashi, Yumiko Iwamoto, Masato Kajikawa, Takeshi Matsumoto, Yasuki Kihara, Kazuaki Chayama, Kensuke Noma, Mitsuo Ochi, Masahiro Nishimura, Koichiro Tsuji, Yukio Kato, Chikara Goto, Yukihito Higashi

**Affiliations:** 1 Department of Cardiovascular Medicine, Hiroshima University Graduate School of Biomedical Sciences, Hiroshima, Japan; 2 Division of Regeneration and Medicine, Hiroshima University Hospital, Hiroshima, Japan; 3 Department of Medicine and Molecular Science, Hiroshima University Graduate School of Biomedical Sciences, Hiroshima, Japan; 4 Department of Cardiovascular Regeneration and Medicine, Research Institute for Radiation Biology and Medicine, Hiroshima University, Hiroshima, Japan; 5 Department of Orthopedic Surgery, Hiroshima University Graduate School of Biomedical Sciences, Hiroshima, Japan; 6 Department of Prosthetic Dentistry, Graduate School of Biomedical Sciences, Nagasaki University, Nagasaki, Japan; 7 Two Cells Co. Ltd, Hiroshima, Japan; 8 Department of Dental and Medical Biochemistry, Hiroshima University Graduate School of Biomedical Sciences, Hiroshima, Japan; 9 Hirohsima International University, Hiroshima, Japan; University of Bristol, United Kingdom

## Abstract

**Background:**

The purpose of this study was to determine whether autologous mesenchymal stem cells (MSCs) implantation improves endothelial dysfunction in a rabbit ischemic limb model.

**Methods:**

We evaluated the effect of MSC implantation on limb blood flow (LBF) responses to acetylcholine (ACh), an endothelium-dependent vasodilator, and sodium nitroprusside (SNP), an endothelium-independent vasodilator, in rabbits with limb ischemia in which cultured MSCs were implanted (n = 20) or saline was injected as a control group (n = 20). LBF was measured using an electromagnetic flowmeter. A total of 10^6^ MSCs were implanted into each ischemic limb.

**Results:**

Histological sections of ischemic muscle showed that capillary index (capillary/muscle fiber) was greater in the MSC implantation group than in the control group. Laser Doppler blood perfusion index was significantly increased in the MSC implantation group compared with that in the control group. LBF response to ACh was greater in the MSC group than in the control group. After administration of N*^G^*-nitro-L-arginine, a nitric oxide synthase inhibitor, LBF response to ACh was similar in the MSC implantation group and control group. Vasodilatory effects of SNP in the two groups were similar.

**Conclusions:**

These findings suggest that MSC implantation induces angiogenesis and augments endothelium-dependent vasodilation in a rabbit ischemic model through an increase in nitric oxide production.

## Introduction

Atherosclerotic peripheral artery disease (PAD) is associated with increased cardiovascular morbidity and mortality [1], [2]. In the late stage of PAD, progression of tissue hypoperfusion results in ischemic ulceration and gangrene. Unfortunately, amputation is required in patients with PAD who have no alternative treatment options. Various angiogenic approaches, including cell therapy and gene therapy, have been tried for revascularization of ischemic tissue in animal models of ischemia and in clinical trials. Bone-marrow mononuclear cell (BM-MNC) implantation has been shown to induce therapeutic angiogenesis in both ischemic limb models and patients with limb ischemia [3]–[5]. However, BM-MNC implantation requires harvesting a large amount of BM under general anesthesia, which would be a burden for patients with severe complications, such as myocardial ischemia, heart failure, cerebrovascular disease, and renal failure.

Mesenchymal stem cells (MSCs) have pluripotency and differentiate into osteoblasts, chondrocytes, adipocytes, neurons, skeletal muscle cells, endothelial cells and vascular smooth muscle cells [6]–[11]. MSCs can be easily isolated from BM and can rapidly be expanded ex vivo. Autologous MSCs have advantages over BM-MNCs and embryonic stem cells. The amount of aspirated BM can be markedly reduced, cell implantation can be repeatedly performed, there is no formation of carcinoma such as teratocarcinoma and hemangiosacoma, there is no immune rejection, and there are no ethical problems.

The vascular endothelium is involved in the release of various vasodilators, including nitric oxide (NO), prostacyclin and endothelium-derived hyperpolarizing factor, as well as vasoconstrictors [12], [13]. NO plays an important role in the regulation of vascular tone, inhibition of platelet aggregation, and suppression of smooth muscle cell proliferation [14], [15]. Endothelial dysfunction is the initial step in the pathogenesis of atherosclerosis and plays an important role in the development and maintenance of atherosclerosis [16]. Limb ischemia is associated with endothelial dysfunction [5], [17], [18]. Therefore, it is important to evaluate the vascular function of collateral arteries induced by therapeutic angiogenesis. Recently, we have shown that BM-MNC implantation improves impaired endothelial function in patients with PAD [5]. However, there is no information on the effect of MSC implantation on endothelial function of an ischemic limb.

To determine the effects of MSC implantation on angiogenesis and endothelial function in a rabbit hind limb ischemic model, we evaluated histological, angiographical, and flow-imagical formation of capillary and limb blood flow (LBF) responses to endothelium-dependent vasodilation induced by acetylcholine (ACh) and endothelium-independent vasodilation induced by sodium nitroprusside (SNP).

## Materials and Methods

### Schedule of the Study

**Figure 1:**
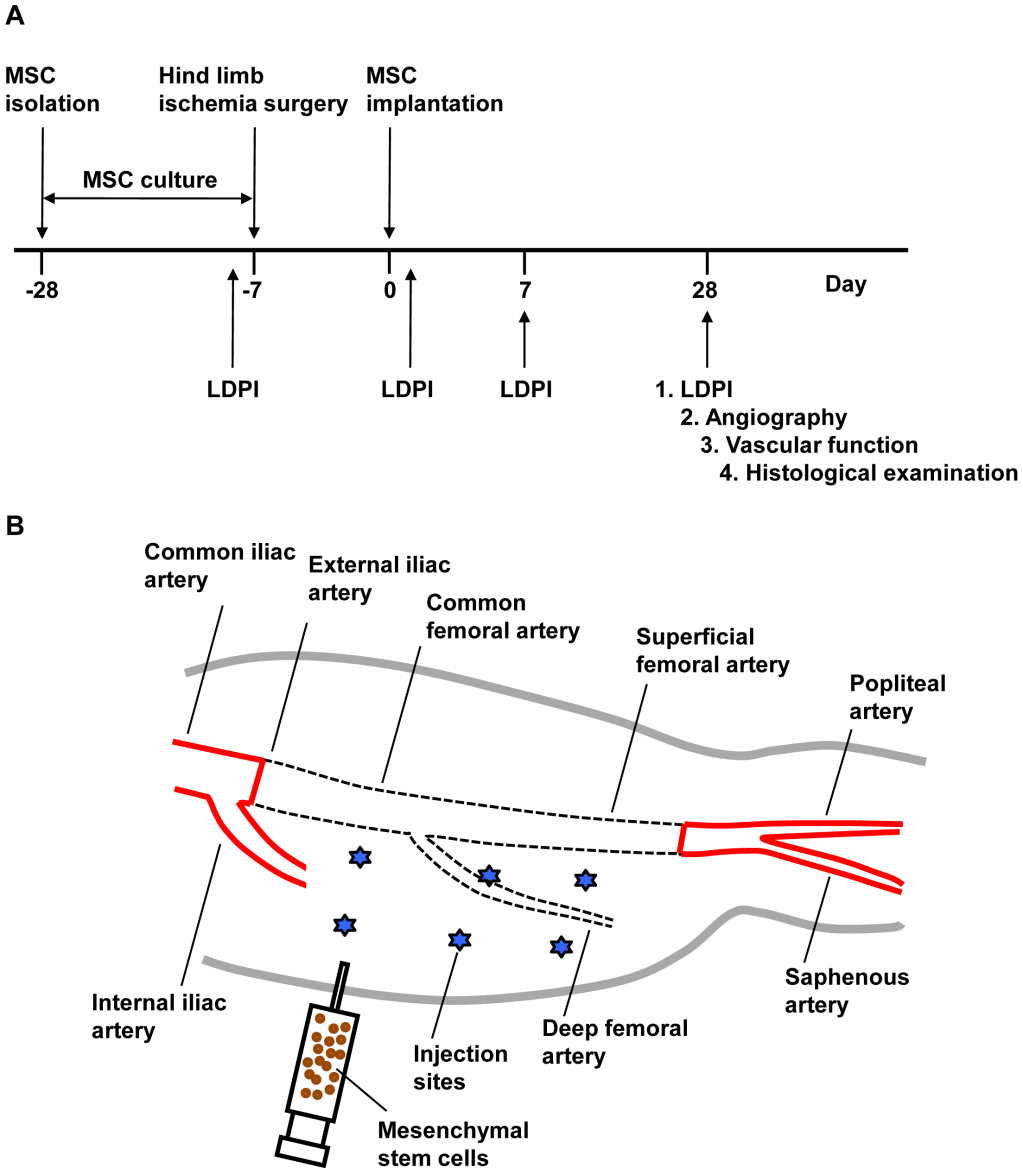
Schedule of the study and hind limb ischemic model. (A) Time schedule of the study, (B) Hind limb ischemic model and MSC injection sites (stars).

Time schedule of the study is shown in Fig. 1A.

### MSC Isolation and Culture

Forty-four male Japanese White rabbits (weight, 3.0–3.5 kg) were used for this study. BM was aspirated from the iliac crest of each rabbit. BM-MNCs were isolated by centrifugation through a Histopaque density gradient (Sigma Chemical Co., St. Louis, Mo) as previously described [19]. To obtain autologous MSC, BM-MNCs were seeded at a density of 0.1 mL aspirate per 35-mm tissue culture dish (Corning, Nogog Park Acton, MA) and maintained in culture medium, 2 mL DMEM (Sigma) containing 10% fetal bovine serum (Hyclone, Logan, UT), 100 U/mL penicillin G (Sigma), and 100 µg streptomycin (Sigma), and incubated at 37°C in a humidified atmosphere containing 5% CO2 and 95% air. Floating cells were removed 3 days after seeding. Then attached cells were fed with fresh culture medium supplemented with 1 ng/mL fibroblast growth factor-2 (FGF-2) (Kaken Pharmaceutical, Tokyo, Japan). Passages were performed when cells were approaching confluence. The cells were seeded at a density of 5×10^3^ cells/cm^2^ in 100-mm culture dishes (Corning) and maintained in 10 mL cultured medium supplemented with 1 ng/mL FGF-2.

### Characteristics of MSCs

In culture medium, MSCs formed a monolayer of adherent cells and looked like long spindle-shaped fibroblastic cells. Adipogenic and osteogenic differentiation was performed by promoting their differentiation into osteocytes and adipocytes with specific differentiation media as described previously [19]. Cell surface antigens on these cells were analyzed by fluorescence activated cell sorting (FACS), and we confirmed these cells were positive for CD29, CD44, CD73, CD90, CD105 and CD166, but negative for CD14 and CD34 as previously described [20].

### Hind Limb Ischemic Model

After confirmation of the number of MSCs needed to be implanted, the rabbits were anesthetized with a mixture of 15 mg/kg ketamine (Sankyo Yell Yakuhin Co., Tokyo, Japan) and 2.0 mg/kg xylazine (Zenyaku Kogyo Co., Kohriyama, Japan) and then under sterile surgical conditions, a longitudinal incision was made on the medial thigh of the left hind limb, extending from the inguinal ligament to a point just proximal to the patella. After the skin incision, the lower limb arterial tree was excised from the origin of the external iliac artery to just proximal to the distal bifurcation of the femoral artery. There was some remaining femoral artery after the excision (Fig. 1B). As a consequence, blood flow to the ischemic limb became completely dependent on collateral vessels arising from the internal iliac artery. The incision was closed in three layers with 3-0 silk. All rabbits were closely monitored by veterinary staff and intra-muscularly received 20 mg/kg gentamycin sulfate (Nitto Medic Co., Toyama, Japan) for 3 days after surgery. The experimental protocols were approved by the Institutional Animal Care and Use Committee of Hiroshima University Graduate School of Biomedical Sciences.

### MSC Implantation

Implantation of cultured autologous MSCs or saline injection was performed under anesthesia 7 days after the hind limb ischemic model had been made. Forty rabbits were randomly divided into an MSC implantation group (n = 20) and a saline injection group (control group, n = 20). In each group 10^6^ autologous MSCs in 1 mL of saline, or 1 mL saline (control) only was injected into the ischemic thigh muscle with a 26-gauge needle at six different points (Fig. 1B). In a previous study, 5×10^6^ bone marrow mononuclear cells, including MSCs, were implanted into the ischemic tissues [3]. In a preliminary study, we confirmed that implantation of 10^5^ MSCs induced angiogenesis and improved endothelial function. Therefore, we selected the number of 10^6^ MSCs. It is expected that this number of MSCs is directly applicable to implantation of human MSCs.

### Differentiation of Implanted MSCs into Endothelial Cells

An additional 4 rabbits were used to examine whether transplanted MSCs differentiate into endothelial cells in ischemic muscle. Before implantation, MSCs were electroporated with a plasmid vector encoding the enhanced green fluorescent protein gene (EGFP) using a microporator (MP-100, PEQLAB Biotechnologie GmbH, Erlangen, Germany) according to the manufacturer’s instructions. After incubation for 24 hours, EGFP-positive cells were sorted by FACSAria (Becton Dickinson Biosciences, Franklin Lakes, New Jersey), and then collected cells (1×10^6^ cells per animal) were transplanted into the ischemic thigh muscle. MSCs tranfected with EGFP were analyzed by flow cytometry (Becton Dickinson Biosciences) for expression of EGFP 24 hours after EGFP transfection. Transfection efficiency of EGFP was >60%. Cell viability was >90%. The cumulative numbers of EGFP-transfected MSCs and non-transfected MSCs after plating were similar (data not shown). The rabbits were sacrificed 28 days after MSC implantation, and then the hind limbs were dissected and samples of the adductor muscle and the semimembranous muscle were removed for histological evaluation. The tissues were placed in plastic cassettes and covered with OCT compound (Tissue-Tek, Sakura Finetechnical Co, Tokyo, Japan) before being snap-frozen in liquid nitrogen. Multiple frozen sections were cut (5 µm in thickness) on a cryostat (CM 3050, Leica-Camera Co., Tokyo, Japan) and placed on glass slides. Direct observation of EGFP-transfected MSCs in thigh muscle was performed with microscopes (model BZ-9000, KEYENCE Co., Tokyo, Japan). Fluorescence resonance energy transfer measurements were performed with a microscope controlled by a BZ II-Analyzer (KEYENCE Co.). Identification of endothelial cells was performed by staining for CD31 with a chicken anti-rat monoclonal antibody against CD31 (Molecular Probes, Inc., Eugene, Oregon, USA.). Cell nuclei were counterstained with 4′,6-diamino-phenylidole (DAPI, Sigma-Aldrich Japan, Inc., Tokyo, Japan).

### Angiography

The collateral arteries in the medial thigh region of the ischemic limb originate from the internal iliac artery. Digital subtraction angiography (Model Advantx-LC-DLX, General Electronic Co., Fairfield, CT) of the hind limb was performed on postoperative day 28 to obtain anatomical measurements of the growth and development of the larger conduit vessels in the medial portion of the upper limb. Each rabbit was anesthetized and the right common carotid artery was exposed. A 4-F polyethylene catheter (Cordis Co., Miami Lakes, FL) was introduced into the right common carotid artery through a small cutdown and the tip of the catheter was positioned just proximal to the point where it bifurcates into the right and left common iliac arteries under fluoroscopic guidance. Perfusion of the hind limb was observed on a monitor in real time and a series of angiographic images were taken after the start of contrast media infusion. Quantitative angiographic analysis of collateral vessel development was performed as follows. A composite of 5 mm^2^ grids was placed over the medial thigh area of a 4-s angiogram. The total number of grid intersections in the medial thigh area, as well as the total number of intersections crossed by a contrast-opacified artery, were counted individually by a single observer blinded to the treatment regimen. An angiographic score was calculated for each film as the ratio of grid intersections crossed by opacified arteries divided by the total number of grid intersections in the medial thigh.

### Measurement of Laser Doppler Perfusion Image

We measured serial LBF using a laser Doppler perfusion image (LDPI) analyzer (Inframeter, Soft Care Co., Iizuka, Japan). Excess hair was removed from the hind limbs. Before initiating scanning, rabbits were placed on a heating plate at 37°C to minimize variations in temperature. The LDPI uses a 12-mW helium–neon laser beam that sequentially scans a 18×12 cm surface area. As the scanning is performed, moving blood cells shift the frequency of incident light according to the Doppler principle. A photodiode collects the backscattered light, and the original light intensity variations are transformed into voltage variations in the range of 0–10 V. A perfusion output value of 0 V was calibrated as 0% perfusion, whereas 10 V was calibrated as 100%. Upon termination of scanning, a colorcoded image representing blood flow distribution is displayed on a monitor. The perfusion signal is subdivided into six different intervals, each displayed as a separate color. Low or no perfusion is displayed as dark blue, whereas the highest perfusion interval is displayed as red. The stored perfusion values behind the color-coded pixels remain available for data analysis. For each time point described, we used LDPI to perform two consecutive measurements over the same region of interest and found little or no difference between the two scans. Accordingly, after recording laser Doppler color images twice, the average perfusions of the ischemic and nonischemic limbs were calculated on the basis of colored histogram pixels. To minimize variables, including ambient light and temperature, calculated perfusion was expressed as the ratio of ischemic to nonischemic hindlimb perfusion. Perfusion analyses were performed sequentially before hind limb ischemic surgery and after MSC implantation or saline injection at day 1, day 7 and day 28 under anesthesia.

### Histological Determination of Capillary Density

After angiography and examination of vascular response, the rabbits were immediately sacrificed and then the hind limbs were dissected and samples of the adductor muscle and the semimembranous muscle were removed for histological evaluation 28 days after MSC implantation or control saline injection. These two muscles were chosen because they are the two principal muscles of the medial thigh, and each was originally perfused by the deep femoral artery, which was ligated when the common/superficial femoral arteries were excised. The tissues were placed in plastic cassettes and covered with OCT compound (Tissue-Tek, Sakura Finetechnical Co.) before being snap-frozen in liquid nitrogen. Multiple frozen sections were cut (5 µm in thickness) on a cryostat (CM 3050, Leica-Camera) and placed on glass slides. Tissue sections were stained for alkaline phosphatase (AP) using the indoxyl-tetrazolium method to detect capillary endothelial cells. AP-positive capillary endothelial cells were counted under a light microscope (×200) to determine the capillary density. Five fields from each sample were randomly selected for the counts. To ensure that the capillary density was not overestimated as a consequence of myocyte atrophy or underestimated because of interstitial edema, the capillary/muscle fiber ratio was also determined.

### Measurement of LBF

LBF was measured using an electromagnetic flowmeter (MFV-3000, Nihon Koden Co., Tokyo, Japan). Limb vascular responses to ACh (Daiichi Pharmaceutical Co., Tokyo, Japan) and SNP (Maruishi Pharmaceutical Co., Osaka, Japan) were evaluated after angiography at day 28 in the MSC group and control group. Rabbits were kept in the supine position in an air-conditioned room (temperature, 22°C to 25°C) under anesthesia throughout the study. After each rabbit had spent 15 minutes in the supine position, baseline LBF was measured. Then LBF responses to ACh and SNP infusion were measured. ACh (100, 300 and 1000 µg/kg/min) and SNP (10, 30 and 100 µg/kg/min) were infused intra-arterially for 5 minutes at each dose using a 4-F polyethylene catheter (Cordis Co.) that was inserted into the femoral artery in the ischemic limb in which MSCs had been implanted or saline had been injected. To avoid drug infusion order bias, infusions of ACh, an endothelium-dependent vasodilator, and SNP, an endothelium-independent vasodilator, were carried out in random order. Each study proceeded after the LBF had returned to baseline. To examine the effect of MSCs on release of NO, we measured LBF in the presence of the endothelial NO synthase (eNOS) inhibitor N^G^-nitro-L-arginine (L-NA, Sigma). The responses of leg vasculature to ACh after intra-arterial co-infusion of L-NA (8 µmol/kg/min) were evaluated.

### Statistical Analysis

Results are presented as means±SD. All reported P values were two-tailed. Values of P<0.05 were considered significant. Comparison between 2 groups was made using Student’s *t* test. Comparisons of time course curves of parameters were analyzed by two-way analysis of variance for repeated measures on one factor followed by Bonferroni correction for multiple-paired comparisons.

## Results

### MSC Culture

**Figure 2:**
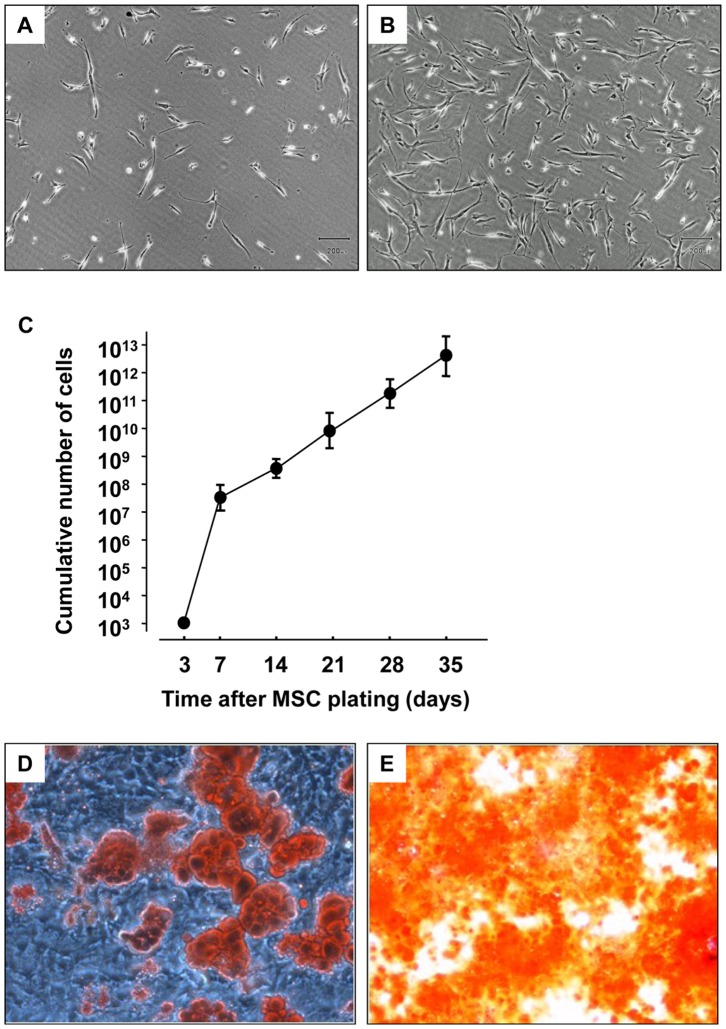
MSC culture and the ability of adipogenic and osteogenic differentiation. (A and B) Morphological appearance of rabbit MSC, (A) At day 5 after initial plating, adherent cells were spindle-shaped, (B) At day 10 after initial plating, cells had grown to semiconfluence (Original magnification: ×100), (C) Line graph shows the relationship between cumulative number of cells and days after MSC plating (n = 5), (D) Adipogenic differentiation was confirmed by staining with Oil Red-O to visualize neutral lipid accumulation (Original magnification: ×20), (E) Osteogenic differentiation was confirmed by staining with Alizarin Red S (Original magnification: ×40).

Adherent spindle-shaped cells were seen in the cultured dish at about 4 to 5 days after initial plating (Fig. 2A). These cells rapidly reached semiconfluence in the culture medium at about 8 to 12 days after initial plating (Fig. 2B). The MSC population expanded from 10^3^ cells to over 10^6^ cells at 4 to 5 passages. The cumulative number of MSCs increased linearly from 5.1×10^8^±3.2×10^8^ to 3.0×10^12^±1.8×10^12^ at day 35 (Fig. 2C). The ability of adipogenic and osteogenic differentiation was confirmed by promoting their differentiation into osteocytes and adipocytes with specific differentiation media. Adipogenic differentiation was revealed after 8 days by staining with Oil Red-O to visualize neutral lipid accumulation (Fig. 2D). Osteogenic differentiation was confirmed after 2 weeks by staining with Alizarin Red S (Fig. 1E). MSCs do not have a specific antigen profile. However, we confirmed that isolated cells were negative for typical hematopoetic antigens CD34, CD38 and CD45 and were positive for molecular markers (e.g., BMP4, IGF1, LIF and PRG1) by using a real-time PCR as previously described.^19.^

### Angiographic Score after Implantation of MSCs

**Figure 3:**
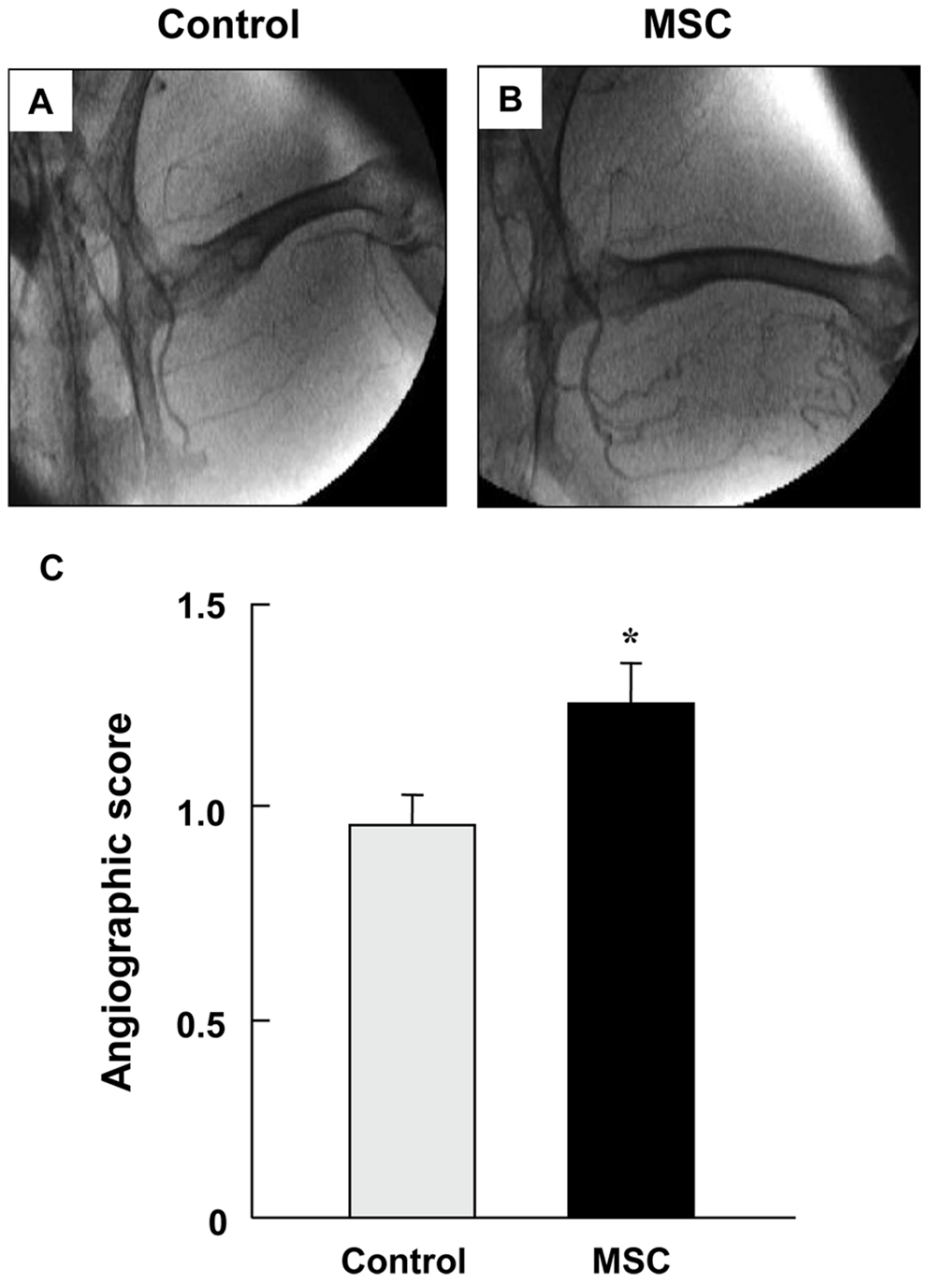
Angiographic Score after Implantation of MSCs. (A and B) Representative angiography at 28 days after MSC implantation or saline injection (control) in rabbits with limb ischemia, (C) Angiographic score at 28 days after MSC implantation or saline injection (control) in rabbits with limb ischemia. *P<0.05 vs. the control group.

At day 28 after MSC implantation, marked formation of new collateral vessels in the lower limbs were seen (Fig. 3B), whereas angiography showed poor collateral vessel formation in lower limbs of the control group (Fig. 3A). Angiographic score at day 28 after MSC implantation was significantly higher in the MSC group than in the control group (1.25±0.11 vs. 0.96±0.08, P<0.01) (Fig. 3C).

### LDPI before and after Implantation of MSCs

**Figure 4:**
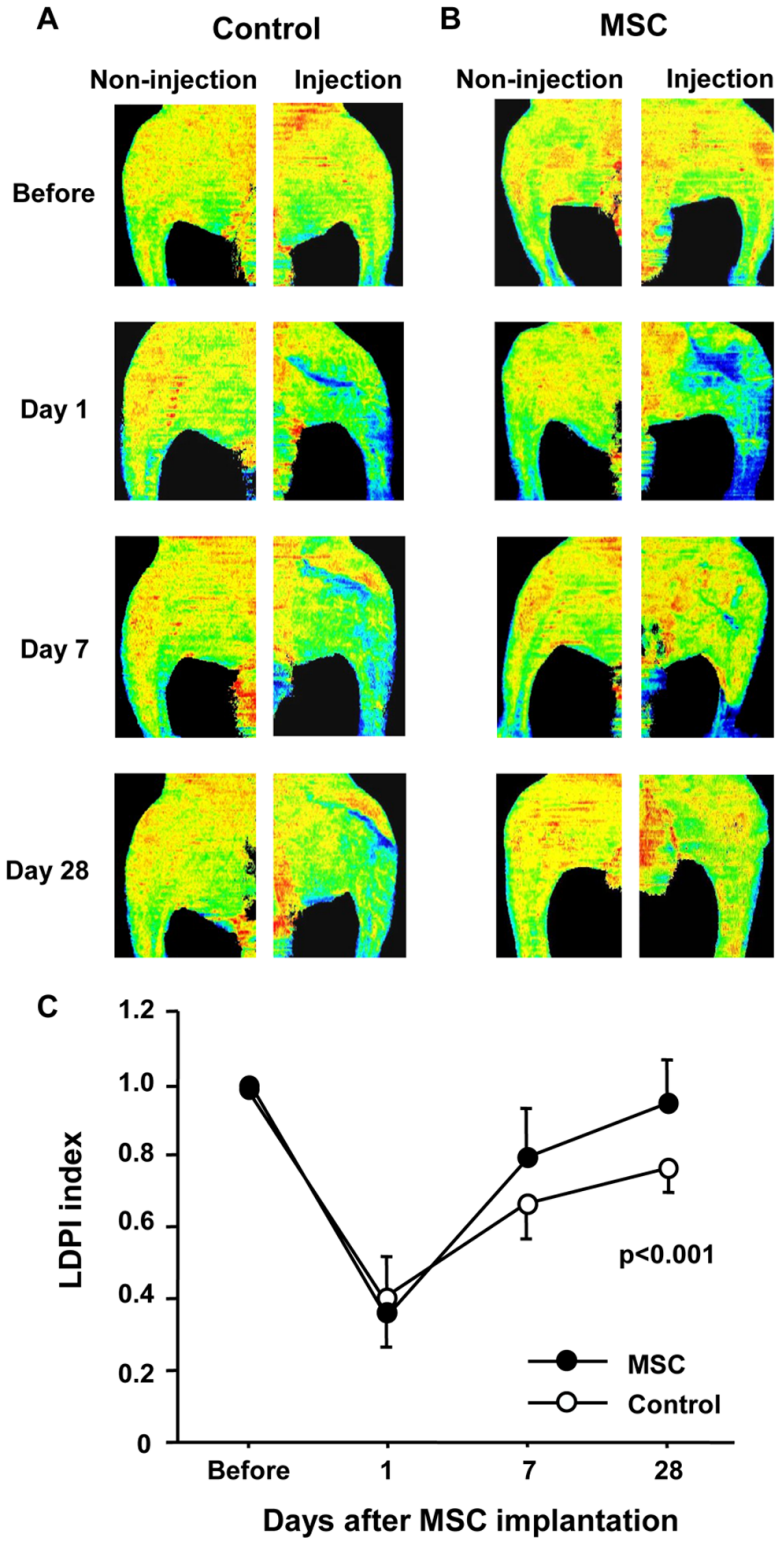
LDPI after Implantation of MSCs. (A and B) Representative laser LDPIs before and at 1, 7 and 28 days after MSC implantation or saline injection (control) in rabbits with limb ischemia, (C) High perfusion is indicated by red, and low perfusion is indicated by blue. Time-dependent change in LDPI index at 1, 7 and 28 days after MSC implantation or saline injection (control).

LDPIs before and at 1, 7 and 28 days after implantation of MSCs and saline injection are shown in Fig. 4A and 4B. Quantitative analysis of LDPI revealed an increase in blood flow after the implantation of MSCs in ischemic hindlimbs in comparison with blood flow in control hindlimbs that received injection of saline. Time-dependent change in LDPI index after implantation of MSCs or saline injection was significantly greater in the MSC group than in the control group (P<0.001) (Fig. 4C).

### Macroscopic Findings after Implantation of MSCs

One necrotic toe in the MSC-implanted group and 4 necrotic toes in the control group during the follow-up period were observed. There was no significant difference between the number of necrotic toes in the MSC-implanted group and that in the control group (P = 0.15).

### Histological Determination of Capillary Density after Implantation of MSCs

**Figure 5:**
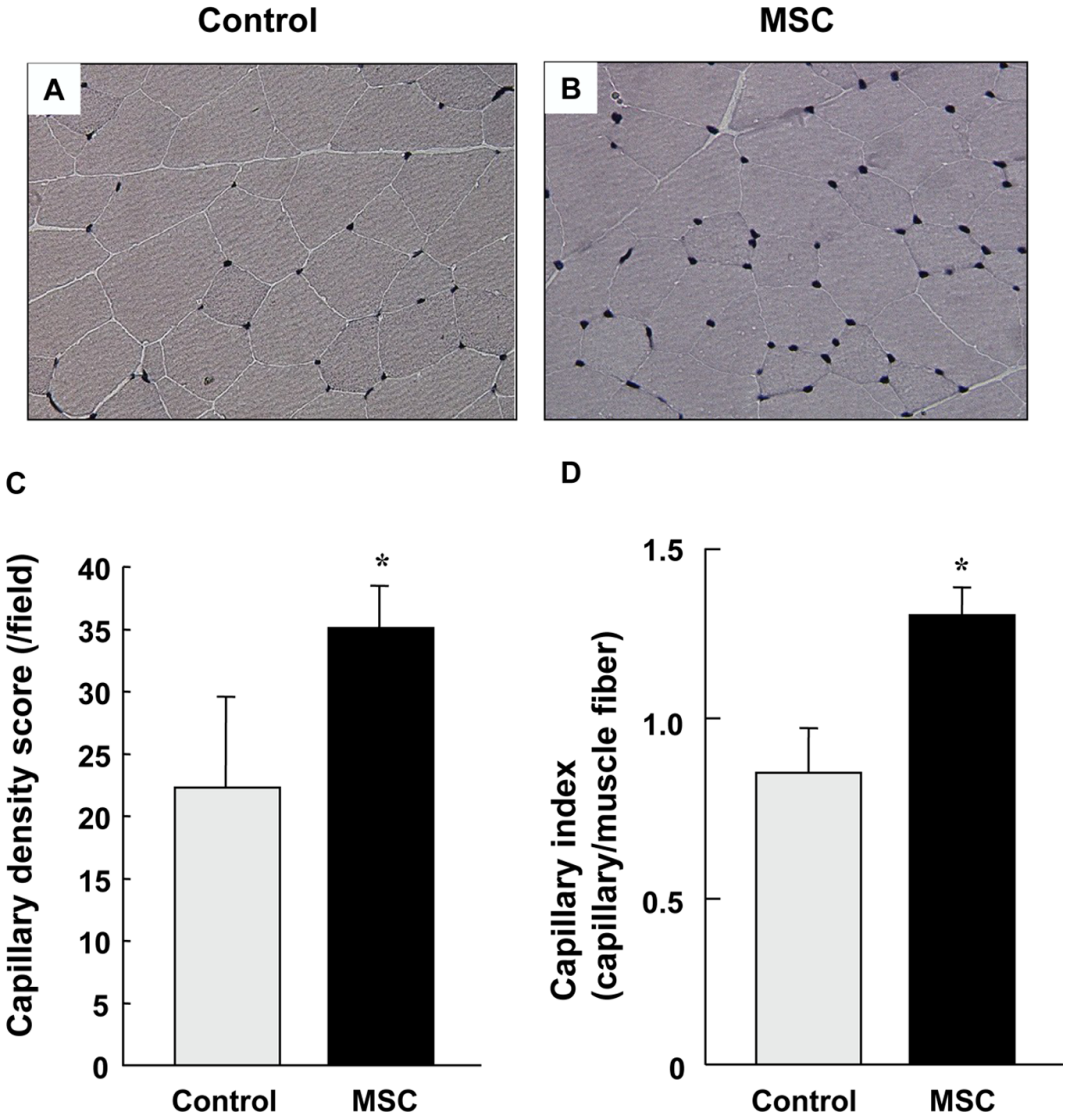
Histological Determination of Capillary Density after Implantation of MSCs. (A and B) Representative alkaline phosphatase-stained cells at 28 days after MSC implantation or saline injection (control) in rabbits with limb ischemia. (Original magnification: ×200), (C and D) Capillary density score and capillary index at 28 days after MSC implantation or saline injection (control) in rabbits with limb ischemia. *P<0.05 vs. the control group.

A large number of capillaries were detected in the ischemic muscle of the MSC group compared with that in the control group at day 28 after MSC implantation or saline injection (Fig. 5A and 5B). Both capillary density score and capillary/muscle fiber ratio of the ischemic hindlimb were significantly higher in the MSC group than in the control group (35.1±3.7 vs. 22.2±7.8 and 1.27±0.08 vs. 0.71±0.22, P<0.001, respectively) (Fig. 4C and D).

### Differentiation of Implanted MSCs into Endothelial Cells

**Figure 6:**
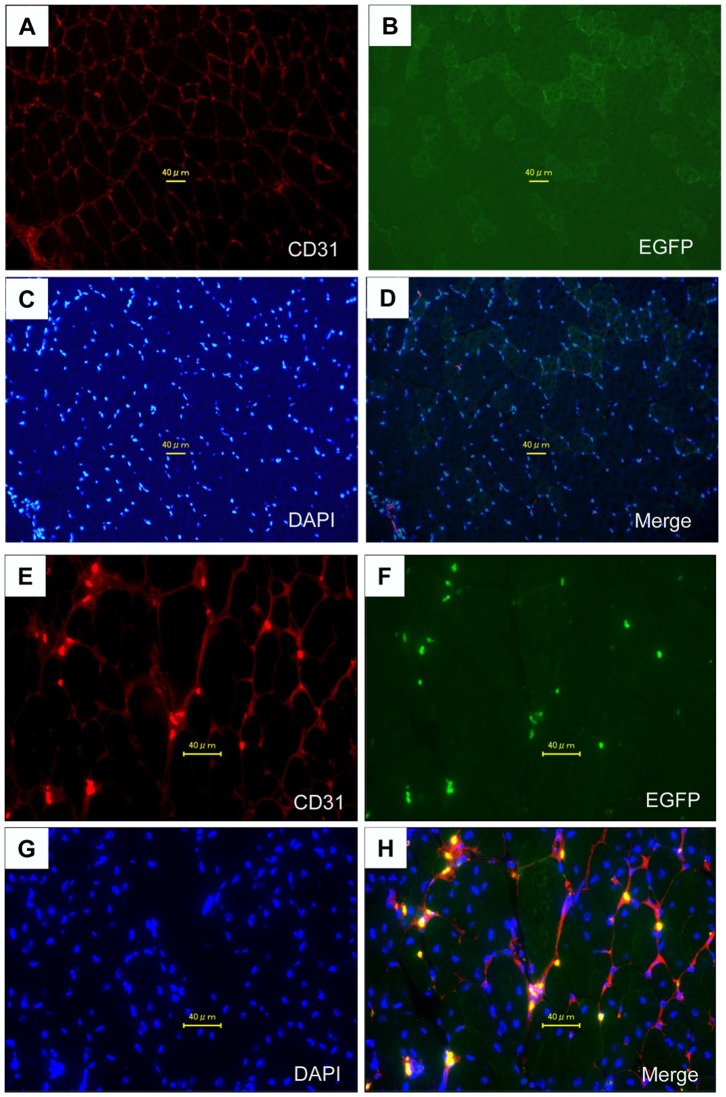
Differentiation of Implanted MSCs into Endothelial Cells. (A and E) Representative CD31-stained cells as red color, (B, F) EGFP-transfected cells as green color, (C and G) DAPI-labeled cell nuclei as blue color, (D and H) CD31/EGFP-double-positive cells as yellow color at 28 days after MSC implantation in most of the ischemic limb tissues (top) and the severely injured areas (bottom) in rabbits with limb ischemia.

Immunochemical staining of CD31 revealed the presence of capillary endothelial cells in ischemic limb tissues at day 28 after MSC implantation (Figs. 6A and 6D). EGFP-positive cells were not detected in most of the ischemic limb tissues (Fig. 6B). In addition, capillary sprouting involving CD31/EGFP merged cells was absent in the relatively intact area in ischemic tissues (Fig. 6D). In very few fields in ischemic limb tissues (1 per about 2000 slices of samples of the adductor muscle and semimembranous muscle), EGFP-positive and CD31/EGFP merged cells were detected in the endothelial capillary structure at day 28 after MSC implantation (Fig. 6E, 6F and 6H). These merged cells were present near and within necrotic or scar lesions in ischemic limb tissues. EGFP-positive and CD31/EGFP merged cells were not detected in ischemic limb tissues in 1 of the 4 rabbits, while CD31-positive cells were detected in the endothelial capillary structure in all rabbits that had effective angiogenesis by MSC implantation.

### LBF Responses to ACh and SNP after Implantation of MSCs

**Figure 7:**
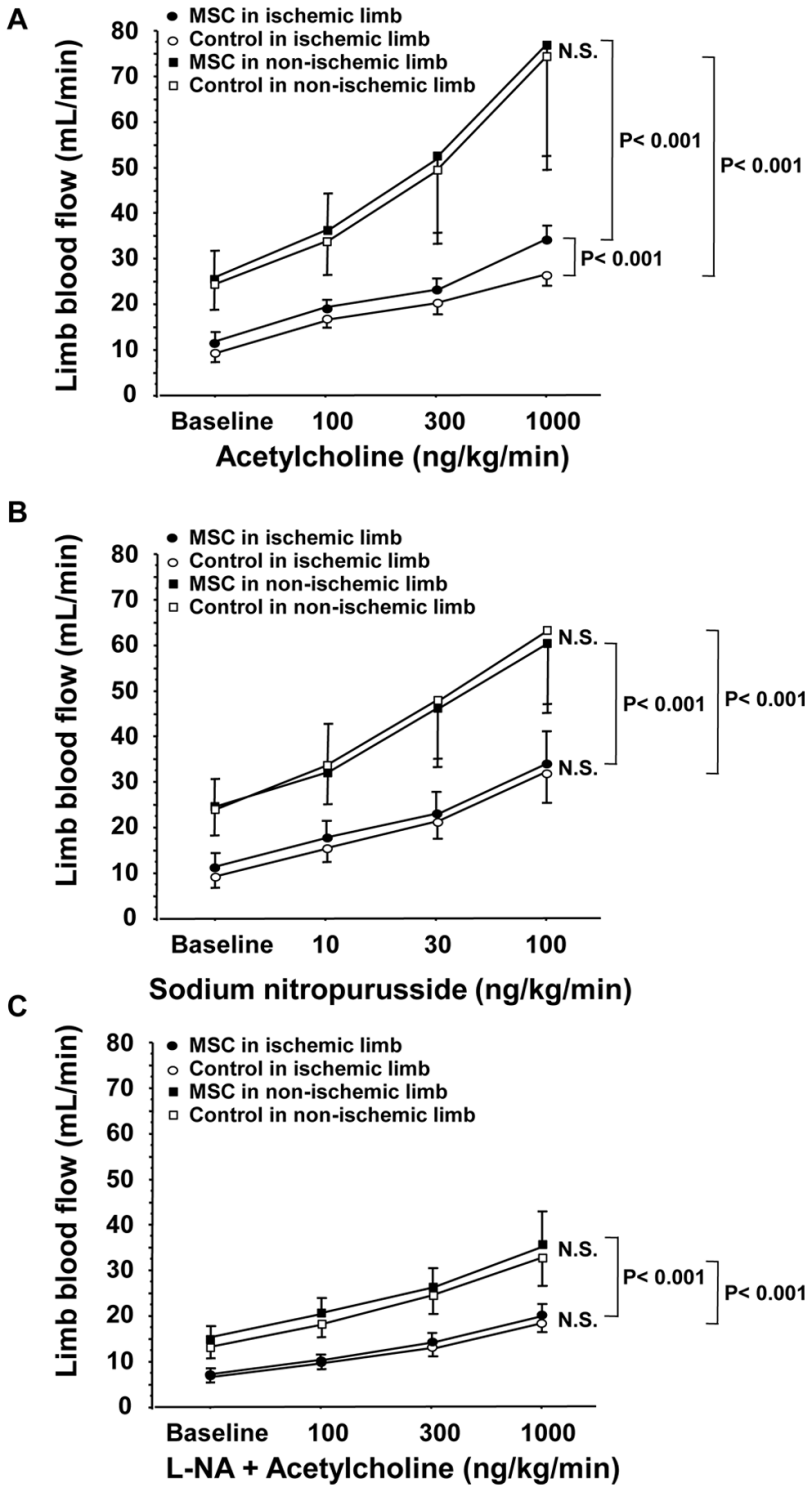
Vascular function after Implantation of MSCs. (A) Comparison of limb blood flow responses to acetylcholine administration, (B) sodium nitroprusside administration and (C) acetylcholine administration in the presence of N^G^-nitro-L-arginine (L-NA) at 28 days after MSC implantation or saline injection (control).

Baseline LBF tended to be higher, but not significantly higher, in the MSC group compared with that in the control group at the end of the 28-day study period (12.0±2.1 vs. 9.2±1.7 mL/min, P = 0.08). Baseline LBF was significantly lower in the ischemic limb than in the non-ischemic (MSC group: 12.0±2.1 vs. 26.1±6.9 mL/min, P<0.001; Control group: 9.2±1.7 vs. 24.8±6.8 mL/min, P<0.001). The response of LBF to infusion of ACh was greater in the MSC group than in the control group at the end of the 28-day study period (P<0.001) (Fig. 7A). The increases in LBF during infusion of SNP were similar at the end of the 28-day study period in the MSC group and the control group (Fig. 7B). In the both MSC group and control group, dose response curves to ACh and SNP were significantly decreased in the ischemic limb compared to those in the non-ischemic during the follow-up period (Fig. 7A and 7B). No significant change was observed in arterial blood pressure or heart rate with intra-arterial infusion of ACh and SNP.

Intra-arterial infusion of L-NA significantly decreased baseline LBF from 11.9±2.5 to 7.4±0.6 mL/min (P<0.05) in the ischemic limb and from 25.9±6.7 to 15.0±2.5 mL/min (P<0.01) in the non-ischemic limb in the MSC group and from 9.4±2.3 to 7.1±0.5 mL/min (P<0.05) in the ischemic limb and from 25.5±6.9 to 13.1±2.4 mL/min (P<0.01) in the non-ischemic limb in the control group. Changes in basal limb vascular responses to L-NA were similar in both the ischemic and non-ischemic limbs in the two groups (Fig. 7C). In both the MSC group and control group, intra-arterial infusion of L-NA decreased the LBF response to ACh in both the ischemic and non-ischemic limbs. After the intra-arterial infusion of L-NA, there was no significant difference between LBF responses to ACh in the MSC group and control group (Fig. 7C). In both the MSC group and control group, dose response curves to ACh in the presence of L-NA were significantly decreased in the ischemic limbs compared to those in the non-ischemic limbs during the follow-up period (Fig. 7C). Neither arterial blood pressure nor heart rate was significantly changed by intra-arterial infusion of ACh in the presence of L-NA.

## Discussion

In the present study, autologous MSC implantation improved endothelium-dependent vasodilation in rabbits with limb ischemia. The eNOS inhibitor L-NA abolished the augmentation of endothelium-dependent vasodilation, suggesting that MSC implantation-induced improvement in endothelial function was due to an increase in NO release. However, MSC implantation-induced improvement in endothelial function did not reach the grade of endothelial function in the non-ischemic limb. MSC implantation did not alter SNP-induced vasodilation. This beneficial effect of MSC implantation on vascular function may be selective in endothelium-dependent vasodilation (endothelial cell function) but not in endothelium-independent vasodilation (smooth muscle cell function).

In clinical setting, it is important to enable to perform the autologous cells implantation to avoid severe adverse effects, such as graft-versus-host disease. In a preliminary study, we tired to aspirate BM and implant autologous MSCs in various animal ischemic hindlimb models. The minimum size of animal that we could perform autologous MSC implantation and examine the vascular responses to vasoactive agents was the rabbit. Therefore, we selected a rabbit ischemic hindlimb model.

Autologous MSCs have several advantages over BM-MNCs and embryonic stem cells: 1) the amount of aspirated or collection of BM can be markedly reduced because the number of MSCs can be rapidly increased (Fig. 2C). (In regimes of BM-MNC implantation, about 500 to 700 mL of BM is aspirated from the ileum under general anesthesia.), 2) MSCs can be banked after cell culture, and cell implantation can be repeatedly performed, 3) there is no formation of carcinoma such as teratocarcinoma and hemangiosarcoma, which can develop with direct embryonic stem cell implantation, and 4) there are no ethical problems. In addition, several studies have shown that MSCs per se have immunoregulatory properties [21]–[26]. Allogenic MSCs have recently been shown to suppress T cell proliferation in vitro and in vivo [21]–[23]. Indeed, administration of MSCs safely allow transplantation of donor cells into conditioned recipients without the use of pharmacologic immunosuppression [24]. Co-infusion of allogenic human MSCs and donor cells can prevent or treat acute-graft-versus-host disease in patients who have undergone BM transplantation [25], [26]. These findings suggest the possibility of using allogenic MSC implantation for angiogenesis.

In the present study, MSC implantation increased angiographic score, LDPI index, and capillary density score and capillary index, although basal LBF measured by an electromagnetic flowmeter did not increase significantly (P = 0.08) after MSC implantation. Therefore, one possible mechanism by which MSC implantation augments endothelium-dependent vasodilation is by increasing shear stress resulting from blood flow. Acute or chronic increases in shear stress potently stimulate the release of NO in isolated vessels [27] and cultured cells [28]. Sessa et al. [29] demonstrated that the increase in shear stress in epicardial coronary arteries enhanced the expression of the vascular eNOS gene, leading to ACh-stimulated NO release. Upregulation of eNOS mRNA levels and eNOS protein levels by increase in shear stress after MSC implantation may contribute to improvement in endothelial function through an increase in NO production. Several lines of evidence have shown that putative shear stress-mediated mechanotranductions, such as the Ras/Raf/MEK/ERK pathway, c-Src, PI3K/Akt, HSP and HIF-1, contribute to the upregulation of eNOS mRNA and eNOS protein, leading to an increase in NO production [30], [31]. Shear stress is sensed and transduced into biochemical signals by multiple pathways in the vasculature, resulting in various biological responses, including increase in eNOS activity.

MSCs include various angiogentic cytokines, such as vascular endothelial growth factor (VEGF) and angiopoietin families, and release these factors [32]. VEGF induces the formation of collateral vessels and increases collateral blood flow, leading to improvement in endothelium-dependent vasodilation [33]. In addition, VEGF directly up-regulates eNOS expression and increases subsequent NO release [34]. Rajagoplana et al. [18] have recently reported that gene therapy using an adenoviral vector encoding a 121-amino-acid isoform of VEGF augmented ACh-induced vasodilation in lower leg circulation in patients with PAD. These findings suggest that increased VEGF protein levels contribute to angiogenesis with MSC implantation. In addition, Fontana et al. [35] reported that VEGF stimulates the recruitment of HSP90- and PI3K/Akt-dependent eNOS phosphorylation, leading to an increase in NO production. It is likely that VEGF released from MSC contributes to the angiogenesis-induced improvement in endothelium-dependent vasodilation.

Although the mechanism by which MSC implantation improves endothelial function in rabbits with limb ischemia is not clear, the multiplier effect of endothelial cell lineage may contribute to the angiogenesis-induced improvement in endothelium-dependent vasodilation. It is controversial whether adult stem cells can differentiate into the endothelial cells or endothelial progenitor cells in vivo. Results of recent studies have shown that MSCs easily differentiate into osteoblasts, chondrocytes, and adipocytes in vitro and in vivo [6]–[9]. Endothelial cells that are derived from the mesenchyme belong to the mesenchymal tissues. However, we are not able to differentiate MSCs derived from humans, rabbits, or rats into an endothelial cell lineage *in vitro* under several conditions (data not shown). Some studies have demonstrated the possibility that adult BM cells, including MSCs, can differentiate into vascular endothelial cells or endothelial progenitor cells in a hindlimb ischemic model and in a heart ischemic model [10], [11]. However, Ziegelhoeffer et al. [36] have recently shown that although implantation of BM-derived cells increases collateral vessel formation in an ischemic limb model, these cells are not incorporated into the growing vasculature, suggesting that angiogenesis with adult cells is due to several angiogenic growth factors and cytokines released by implanted cells. In addition, O’Neill et al. [37] have shown that mobilization of bone marrow-derived cells contributes to angiogenesis in response to hypoxia without transdifferentiation into endothelial cells. Results of several studies support the concept that angiogenic growth factors and cytokines released by implanted cells predominately promote angiogenesis [38], [39]. It has been reported that implanted GFP-positive cells accumulate around growing collateral arteries, while GFP-positive BM cells do not differentiate into vascular smooth muscle cells and endothelial cells in a mice hindlimb ischemic model [36]. In the present study also, capillary sprouting involving GFP/CD31-double-positive cells was absent in most of the ischemic limb tissues with transplanted MSCs. Interestingly, we found a capillary containing a very small number of GFP/CD31-double-positive cells in a severely injured tissue area. These findings suggest that the mechanisms of angiogenesis induced by MSC implantation may be different in the ischemic area or ischemic condition. A large part of MSC implantation-induced angiogenesis should be due to mobilization of endothelial progenitor cells by angiogenic growth factors. It is likely that a part of the transdifferentiation of MSCs into endothelial cells is involved in angiogenesis in severely injured areas, although the degree of contribution to angiogensis with transdifferentiation of MSCs is very low.

### Study Limitations

Unfortunately, in the present study, we did not perform angiography in the non-ischemic limbs. Assessment of the non-ischemic limbs by angiography would enable more specific conclusions regarding angiogenesis in the ischemic limbs after MSC implantation to be drawn.

In the present study, we confirmed that infusion of L-NA, an eNOS inhibitor, completely abolished the augmentation of implanted MSC-induced endothelium-dependent vasodilation, suggesting that activation of the eNOS/NO pathway contributed to the improvement in endothelial function in the MSC-implanted group. Direct measurement of eNOS activity, NO and VEGF in the vasculature of the ischemic limb would enable a more specific conclusion regarding the role of the eNOS/NO pathway in improvement in endothelial function after MSC implantation to be drawn.

We confirmed the regions of the adductor muscle and the semimembranous muscle during the skin incision. However, when we injected MSCs into the ischemic limb, the skin incision was closed. Although we believe that the MSCs were implanted into the adductor muscle and semimembranous muscle in the ischemic thigh muscle, we were uncertain about the muscle into which MSCs were injected.

In conclusion, although the effectiveness of therapeutic angiogenesis with angiogenic cytokine gene therapy and with BM-MNC implantation in patients with PAD has been established, MSC implantation therapy may provide a new aspect of therapeutic angiogenesis in such patients. Unfortunately, although the precise mechanisms by which MSC implantation promotes angiogenesis and augments endothelial function are unclear, MSC might be promising in therapeutic angiogenesis. Future studies are needed to determine the role of therapeutic angiogenesis with MSCs in endothelial function and confirm the efficacy, safety and feasibility of MSC implantation for long periods.
